# *N*-Glycoproteins Have a Major Role in MGL Binding to Colorectal Cancer Cell Lines: Associations with Overall Proteome Diversity

**DOI:** 10.3390/ijms21155522

**Published:** 2020-08-01

**Authors:** Martina Pirro, Yassene Mohammed, Sandra J. van Vliet, Yoann Rombouts, Agnese Sciacca, Arnoud H. de Ru, George M. C. Janssen, Rayman T. N. Tjokrodirijo, Manfred Wuhrer, Peter A. van Veelen, Paul J. Hensbergen

**Affiliations:** 1 Center for Proteomics and Metabolomics, Leiden University Medical Center, 2333ZC Leiden, The Netherlands; M.Pirro@lumc.nl (M.P.); y.mohammed@lumc.nl (Y.M.); agnese.sciacca01@universitadipavia.it (A.S.); A.H.de_Ru@lumc.nl (A.H.d.R.); G.M.C.Janssen@lumc.nl (G.M.C.J.); R.T.N.Tjokrodirijo@lumc.nl (R.T.N.T.); m.wuhrer@lumc.nl (M.W.); P.A.van_Veelen@lumc.nl (P.A.v.V.); 2 Department of Molecular Cell Biology and Immunology, Cancer Center Amsterdam, Amsterdam UMC, 1105AZ Amsterdam, The Netherlands; s.vanvliet@vumc.nl; 3 Institut de Pharmacologie et de Biologie Structurale, Université de Toulouse, CNRS, UPS, 31077 Toulouse, France; yoann.rombouts@ipbs.fr

**Keywords:** Glycoproteomics, TMT labeling, C-type lectin, Colorectal cancer, LacdiNAc, Tn antigen

## Abstract

Colorectal cancer (CRC) is the second-leading cause of cancer death worldwide due in part to a high proportion of patients diagnosed at advanced stages of the disease. For this reason, many efforts have been made towards new approaches for early detection and prognosis. Cancer-associated aberrant glycosylation, especially the Tn and STn antigens, can be detected using the macrophage galactose-type C-type lectin (MGL/CLEC10A/CD301), which has been shown to be a promising tool for CRC prognosis. We had recently identified the major MGL-binding glycoproteins in two high-MGL-binding CRC cells lines, HCT116 and HT29. However, we failed to detect the presence of *O*-linked Tn and STn glycans on most CRC glycoproteins recognized by MGL. We therefore investigated here the impact of *N*-linked and *O*-linked glycans carried by these proteins for the binding to MGL. In addition, we performed quantitative proteomics to study the major differences in proteins involved in glycosylation in these cells. Our results showed that *N*-glycans have a significant, previously underestimated, importance in MGL binding to CRC cell lines. Finally, we highlighted both common and cell-specific processes associated with a high-MGL-binding phenotype, such as differential levels of enzymes involved in protein glycosylation, and a transcriptional factor (CDX-2) involved in their regulation.

## 1. Introduction

Glycosylation is one of the most frequent post-translational modifications on proteins and lipids [1]. In eukaryotic cells, protein glycosylation can be mainly grouped into *N*- and *O*-linked glycans, which are synthetized in the endoplasmic reticulum (ER) and/or Golgi apparatus through the sequential/competitive action of numerous glycosyltransferases and glycosidases, encoded by over 200 genes [2]. The expression, activity and subcellular location of these enzymes dictate the overall glycosylation profile within different cells or tissues [1]. Protein glycosylation has a high impact on a wide range of cell biological processes such as proliferation, adhesion, differentiation, cell-cell interactions and immune responses [3]. Accordingly, changes in glycosylation are observed in various diseases, and aberrant glycosylation is recognized for decades as a hallmark of cancer [1,2,4,5,6].

Certain glycans that show increased expression in tumor tissues as compared to normal tissues are referred to as Tumor-Associated Carbohydrate Antigens (TACAs) [7]. Many TACAs are in fact truncated glycan structures resulting from incomplete glycan biosynthesis. One of the most common process is represented by the dysregulation of the initial steps of *O*-glycan biosynthesis mediated by polypeptide α-N-acetylgalactosaminyl-transferases (ppGalNAcTs) that transfer UDP-GalNAc to Ser/Thr residues in a polypeptide, forming Tn antigen (GalNAcα1-Ser/Thr) [6,8]. Humans have 20 genes encoding ppGalNAcTs, and an overexpression, a higher activity and/or a modified subcellular location of these transferases can lead to higher Tn antigen expression, frequently decorating mucins on epithelial tumors [9]. The high levels of GalNAcα2,6-sialyltransferase (ST6GalNAc-I) in tumor tissues gives rise to the Sialyl-Tn epitope (STn, NeuAcα2,6-GalNAcα1-Ser/Thr) and prevents further *O*-glycan elongation. The expression of Tn and STn can also be enhanced by defects in the activity of T-synthase or mutations in its chaperone (Cosmc), which together mediate the synthesis of core 1 *O*-glycans (T antigen, Galβ1,3-GalNAcα1-O-Ser/Thr) [10]. Conversely, some tumors may induce the activation of certain glycosyltransferases involved in the synthesis of neo-antigens decorating glycan termini. For example, the overexpression of fucosyltransferases (Fuc-T 1/3) increases terminal fucosylation and consequently the expression of specific Lewis-blood group antigens (Le^x^, Le^y^). Enhanced expression of sialyltransferases modifies the latter antigens into the sialylated species SLe^x^, and SLe^y^. Higher activity of the mannoside acetyl-glucosaminyltransferase 5 (MGAT5) increases *N*-glycan branching, while dysregulation of the β4-N-acetylgalactosaminyltransferase 3 (B4GALNT3) may lead to the expression of terminal LacdiNAc (GalNAcβ1,4GlcNAcβ1-) on both *N*-linked [11] and *O*-linked glycans [12].

During tumor development, the appearance of TACAs can be detected by cells of the immune system, which can either enhance or dampen the immune response depending on the nature of the interaction [6]. The recognition of TACAs is mediated by glycan binding proteins, i.e., lectins, whose binding specificities depend on their carbohydrate recognition domains (CRD) [9]. Within the large family of lectins, the C-type macrophage galactose-type lectin (MGL/CLEC10/CD301) exclusively recognizes, in a Ca^2+^ dependent manner, terminal GalNAc residues (α- or β-linked) found in the (S)Tn antigens and LacdiNAc epitopes. MGL is primarily expressed by tumor-associated or tolerogenic dendritic cells (DCs) and macrophages. In these cells, ligand binding to MGL activates molecular pathways, resulting in dampening T cell immunity [13]. Besides the primary binding site involved in the recognition of the terminal GalNAc residue, a secondary binding site was found in MGL, which is involved in the binding to the peptide backbone carrying the glycosylated epitope (glycotope) [14]. This suggests that ligand-specific binding may affect different signaling pathways and biological processes.

Since increased expression of MGL ligands is used to discriminate healthy from colorectal cancer (CRC) tissues, and is an independent prognostic marker for CRC stage III patients with lower disease-free survival [15], we recently investigated the identity of cell surface MGL-binding proteins on CRC cells lines [16]. However, due to the relatively low number of identified glycopeptides carrying (S)Tn antigens or LacdiNAc epitope, the underlying differences that could explain the differential MGL binder expression remained unclear. Here our aim was to get a broader understanding of the mechanism behind different MGL-binding phenotypes of these cell lines. For this purpose, we studied the (glyco-)proteins of CRC cell lines using a combination of lectin staining, MGL pull-downs in the absence and presence of PNGase F treatment and overall comparative quantitative proteomics using Tandem Mass Tag (TMT) labeling.

## 2. Results and Discussion

### 2.1. N-Glycans Are Important for MGL-Binding in CRC Cell Lines

**Figure 1:**
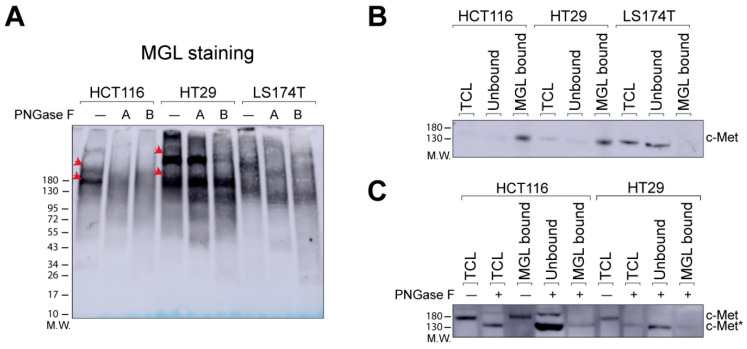
Release of *N*-glycans reduces the MGL-binding of proteins from CRC cell lines. (**A**) MGL pull-downs were performed without (−) or with PNGase F treatment (A and B) and captured proteins were subsequently analyzed by MGL-lectin blot. PNGase F treatment was performed either After (A) or Before (B) performing the MGL pull-down. The lectin blot is a representative of 3 biological replicates (see Figure S1). Red arrows indicate major stained bands. (**B**) MGL-binding of c-Met in HCT116, HT29, and LS174T cells. MGL pull-downs were performed and bound and unbound proteins were analyzed by western blot using a c-Met antibody. TCL: total cell lysate. (**C**) Influence of PNGase F treatment on the binding of c-Met to MGL in HCT116 and HT29 cells. MGL pull-downs were performed with or without prior treatment of the total cell lysate (TCL) with PNGase F. Samples were analyzed by western blot using a c-Met antibody. c-Met* represents the protein with released *N*-glycans. M.W.: Molecular weight.

We recently demonstrated differential binding of the C-type lectin MGL to the colorectal cancer cell lines HCT116, HT29, and LS174T [16]. Although high MGL binding was observed to HCT116 and HT29 cells, binding to LS174T cells was negligible. The comprehensive characterization of *N*- [17] and *O*-glycans [18] expressed by the three CRC cell models used in our study, did not provide an explanation for the difference in MGL binding to these cell lines. We identified the major cell surface proteins binding to MGL in HCT116 and HT29 cells and, for some of these, found a glycopeptide with the MGL specific glycotope (e.g., LacdiNAc (on an *N*-glycan) and Tn antigen), but for many the glycotope remained elusive [16]. To gain more insight into the relative contribution of *N*- and *O*-glycans to MGL binding to glycoproteins of CRC cells, we performed MGL pull-down experiments in combination with PNGase F digestion and lectin (MGL) blots. For this purpose, two different types of experiments were performed. In the first experiment, PNGase F treatment was performed after the MGL pull-down (A, Figure 1A), in the second before the capturing with MGL (B, Figure 1A). As expected, MGL pull-downs with HCT116 and HT29 cells showed a few intense bands corresponding to MGL ligands in the high molecular weight range, which were absent in LS174T cells (Figure 1A, PNGase F untreated (−) samples) in all three biological replicates (Figure 1A and Supplemental Figure S1). With HCT116 cells, these bands disappeared using PNGase F treatment either after (A) or before (B) the MGL pull-down, indicating that the MGL-binding is mainly due to *N*-glycans. On the other hand, PNGase F treatment could not fully abrogate the binding of the major MGL-binding proteins from HT29 cells, even though some major bands disappeared when the cell lysate was treated with PNGase F before the MGL pull-down experiments (B, Figure 1A). These data suggest that *N*-glycans have a substantial role in the binding of proteins to MGL in HCT116 and HT29.

One of the major cell surface MGL-binding proteins in both HT29 and HCT116 cell lines is the receptor tyrosine kinase c-Met [16]. However, in our previous study we could not identify the MGL specific glycotope on the c-Met glycopeptides. Therefore, we also studied here the influence of PNGase F treatment on the binding of c-Met to MGL.

First, using western blot, we could confirm the specific MGL binding of c-Met from HT29 and HCT116 cells (Figure 1B). Importantly, the overall c-Met level in LS174T cells was similar to that in HCT116 and HT29 cells (Supplementary Figure S2A), but in MGL pull-down experiments with LS174T cells, c-Met was found in the unbound fraction (Figure 1B), confirming that in these cells the glycosylation of c-Met is different. Next, we tested the influence of PNGase F treatment on the MGL pull-down of c-Met from HT29 and HCT116 cells. In both cell lines, *N*-glycan release prior to MGL pull-down strongly reduced the MGL-binding of c-Met, which is evident from the high amount of unbound c-Met compared to the bound fraction under this condition (Figure 1C). A concomitant shift in the apparent molecular weight was observed. The fraction of unbound c-Met is negligible without PNGase F treatment (Figure 1B). These results demonstrate that c-Met binding to MGL can, to a large extent, be attributed to *N*-glycans in both HCT116 and HT29 cells.

c-Met is a tyrosine kinase receptor, whose activation is mediated by dimerization following binding to the hepatocyte growth factor (HGF) [19]. This results in phosphorylation of intracellular tyrosines and subsequent activation of downstream pathways involved in cell survival proliferation, migration and invasion [19]. Since HGF-independent hyperactivation of c-Met is involved in the carcinogenesis of CRC, as well as in many other human malignancies [19], we investigated the activation of the receptor in the three CRC cell lines by western blot. This showed c-Met phosphorylation in the two high-MGL-binding cell lines, but not in the low MGL-binding cell line (Supplementary Figure S2B). However, the role of the differential glycosylation in c-Met signaling remains to be determined.

**Figure 2:**
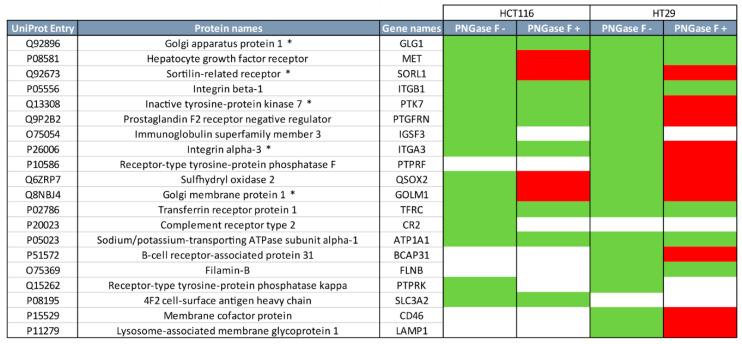
Release of *N*-glycans reduces the MGL-binding of proteins from CRC cell lines. MGL pull-downs were performed after *N*-glycan release of the total protein extracts of HCT116 and HT29 cells. Bound proteins were analyzed by SDS-PAGE, trypsin digestion and LC-MS/MS. The top 20 MGL-binding proteins that we previously identified [16] are shown (in green, PNGase F − (white indicates not identified in that cell line)). After *N*-glycans release (PNGase F +) MGL binders maintained (green) or lost (red) the ability to bind to MGL. *: proteins with previous [16] identification of glycopeptides with a LacdiNAc epitope on an *N*-glycan. See Table S1 for further details.

To obtain a broader understanding on the role of *N*- and *O*-glycans in the binding of proteins to MGL, we also performed MGL pull-down experiments using HCT116 and HT29 cells after *N*-glycan release, followed by LC-MS/MS analysis of the bound proteins. We focused our qualitative comparison on the top 20 MGL-binding proteins that we previously identified [16]. We assigned a protein as an MGL binder when it was identified in two out of three biological replicates. In Figure 2 these are colored in green, while non MGL binders after *N*-glycan release are colored in red. In line with the data shown in Figure 1C, c-Met lost its capability to bind to MGL after *N*-glycan release in HCT116 cells (Figure 2). On the other hand, binding of c-Met to MGL was still observed after PNGase F treatment, in HT29 cells, even though the number of peptides observed was low (Supplementary Table S1). Of note, SORL1, PTK7, and GOLM1, which were previously shown to carry a LacdiNAc epitope on an *N*-glycan in HT29 cells [16], lost the binding to MGL after PNGase F release. On the contrary, proteins such as integrins (ITGB1 and ITGA3) and TFRC still bind to MGL despite the *N*-glycan release. Overall, the MGL binding of more than half of major MGL-binding proteins was affected by *N*-glycan release.

Altogether, our results demonstrate a noteworthy contribution of *N*-glycans to the MGL-binding in CRC cell lines. This can most probably be explained by the presence of the LacdiNAc epitope.

### 2.2. Quantitative Proteomics Provides Insights into Glycosylation Mechanisms Involved in High MGL Binding

We next investigated the potential mechanism(s) responsible for the differential MGL binding to CRC cell lines, such as (i) variable expression of proteins carrying the MGL epitope (ii) alteration in *N-/O*-glycosylation pathways (e.g., levels of glycosyltransferases or transcription factors). Therefore, we performed comparative bottom-up quantitative proteomics. For this purpose, protein extracts from HCT116, HT29, and LS174T cells (three biological replicates for each) were digested with trypsin, isotopically labeled (9-plex TMT labeling (Tandem Mass Tags)), mixed, fractionated and analyzed by LC-MS/MS. Overall, this approach resulted in the identification of 6126 proteins. For 5141 of these, quantitative data was obtained (Table S2).

Binary comparisons of the cell lines showed between 175 and 303 proteins to be differentially abundant (Table S2, Figure S3). Among these, we observed several proteins that have previously been found to be drivers of initiation and progression of cancer. For example, we found higher levels of TP53 in HT29 cells compared to the other two, in line with the reported overexpression of TP53 in this cell line (Table S2) [20].

We next focused on the differences that might explain the differential MGL-binding between these cell lines. First, we checked the relative abundances of the top-20 cell surface MGL-binding proteins that we previously identified [16] (Table S3). In line with the experiments shown above (Figure S2A), the overall proteomics data showed similar levels of c-Met in all three cell lines, irrespective of the MGL binding to these cells (Table S3). This was also true for most of the other major MGL-binding proteins that we previously identified. Hence, the MGL-binding of specific proteins in HT29 and HCT116 cells compared to LS174T cannot solely be explained by the higher levels of these proteins in these cells, even though some differences were observed. For example, HCT116 and HT29 cells have higher levels of the MGL binder ITGA3 in comparison to LS174T.

**Figure 3:**
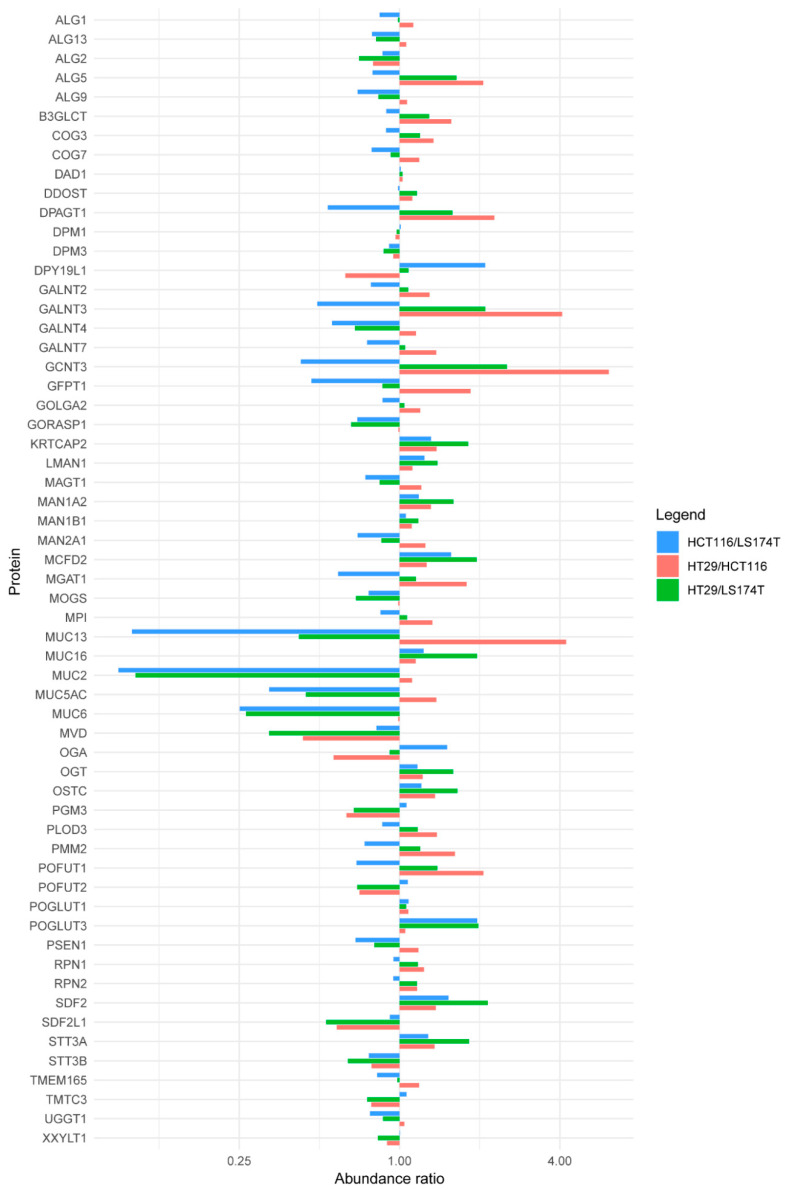
Quantitative proteomics analysis of proteins involved in glycosylation in CRC cell lines. The y-axis shows proteins involved in the glycosylation machinery covered in our study. The x-axis shows the ratio of protein abundance in HCT116 versus LS174T (blue), HT29 versus HCT116 (pink) and HT29 versus LS174T (green).

Next, we compared the levels of proteins involved in *N*- and *O*-glycosylation in our cell lines. We limited the analysis to 245 glycosylation-related proteins as annotated in Gene Ontology (filtering for the term “protein glycosylation”). Overall, we covered 30% of these in our dataset (Figure 3), probably indicating that many others are present, but at very low levels.

The initial step of mucin type *O*-glycosylation is mediated by a family of 20 polypeptide N-acetylgalactosaminyltransferases (GALNTs). Seven of these (GALNT 1–5, 7 and 12) were found in our dataset, four of which could be reliably quantified. Although the levels of GALNT2, 4, and 7 were comparable in the three cell lines, GALNT3 was found at higher levels in HT29 compared to HCT116 and LS174T (Figure 3). This is in line with recent evidence of higher GALNT3 mRNA expression in BRAFV600E mutated cell lines, as HT29, which could be responsible for higher Tn expression in this cell line [21], and the relatively high MGL binding, even after *N*-glycan release (Figure 1A). The elongation of Tn is mediated by T-synthase (C1GALT1) and its chaperone Cosmc (C1GALT1C1). These two proteins were not observed in our dataset. However, their mRNA expression was not reported to be different in BRAFV600E CRC cell lines in the study mentioned above [21]. I-branching (GlcNAcβ1-6Gal-R) of mucin type *O*-glycans is mediated by β1,6-N-acetylglucosamine transferase 3 (GCNT3). The quantitative data we obtained for this enzyme revealed higher levels in HT29 and LS174T, in comparison to HCT116 (Figure 3), in accordance with transcriptomic and glycomic data from literature [18,20]. Another characteristic of HCT116 is the overall low level of fucosylation, associated with a more aggressive phenotype [17,18]. In HCT116, this feature can partially be explained by a deletion of 142 amino acids of the GDP-mannose-4,6-dehydratase (GMDS) involved in GDP-L-fucose synthesis, which may lead to misfolding and degradation of the enzyme [22]. Indeed, GMDS protein levels are much higher in HT29 and LS174T in comparison to HCT116 (Table S2).

In the *N*-glycosylation pathway, most of the identified and quantified proteins involved have comparable levels in the three cell lines (Figure 3). An exception is represented by the Dolichyl-phosphate β-glucosyltransferase (ALG5) and UDP-N-acetylglucosamine-dolichyl-phosphate N-acetylglucosaminephosphotransferase (DPAGT1), both involved in the initial steps of oligosaccharide biosynthesis linked to the dolichol molecule. In fact, the two enzymes were found at higher levels in HT29 and LS174T compared to HCT116 (Figure 3).

Altogether, we observed several differences in enzymes involved in protein glycosylation but they did not provide a clear picture with common differences between high-MGL-binding cells on the one hand (HCT116 and HT29) and the low MGL-binding cell line (LS174T) on the other hand. Obviously, this could be because many proteins involved in protein glycosylation are expressed at low levels and could not be covered within the 6000+ proteins identified here. For example, given the considerable contribution of *N*-glycans for binding to MGL as shown here, and the fact that we previously identified the LacdiNAc epitope on peptides from some of the MGL-binding proteins in the CRC cell lines, it would have been interesting to look at β4-N-acetylgalactosaminyltransferase 3 (B4GALNT3). On the other hand, previous experiments with the cell lines used in our study showed that the mRNA levels of B4GALNT3 were similar in these cells [20].

In addition to enzymes directly involved in protein glycosylation, the selection of proteins also included mucins. Five of these were found in our data (MUC2, MUC5AC, MUC6, MUC13 and MUC16) and, with the exception of MUC16, all were found at lower levels in HT29 and HCT116 compared to LS174T (Figure 3). Mucins have been characterized as MGL-binding proteins in other cancer cells [23,24] and Mucin 1 (MUC1) expressed on colorectal cancer tissues can be recognized by MGL [25]. However, none of these mucins was identified as MGL binder in our previous experiments [16] and our current experiments demonstrate an inverse correlation between the expression of these proteins and the degree of MGL binding.

Interestingly, when we looked more in general for proteins that were different between the high binding cell lines (HT29 and HCT116) and the low MGL-binding cell line (LS174T), we observed the cluster of mucins (Figure S4) in close proximity to the transcription factor CDX-2. Together with CDX-1, this protein acts as a transcription factor that in cooperation with HNF4A and 1A, is involved in the regulation of multiple intestinal specific genes [26], as well as fucosyltransferases [27] and α2,6-sialyltransferase [28]. CDX-1 is very similar to CDX-2, and these proteins share several tryptic peptides, but we did not observe a unique CDX-1 peptide in our dataset. In line with our proteomics data, higher CDX-2 (and CDX-1) mRNA levels were found in highly differentiated cells together with multi-fucosylated *N*- and *O*-glycans, e.g., LS174T [18,29] and mucin 2 is up-regulated by CDX-2 [30]. Hence, our data indicate that downstream targets of CDX-2 (and potentially CDX-1), especially the genes encoding proteins involved in glycosylation, could play a role in the differential binding of MGL but this warrants further investigation.

## 3. Materials and Methods

### 3.1. Cell Lines Culture and Lysis

HCT116 and HT29 were provided by the Department of Surgery of the Leiden University Medical Center (Leiden, the Netherlands), whereas LS174T was obtained from the Amsterdam UMC (Amsterdam, the Netherlands). Cell line authentication was performed using short-tandem repeat (STR) profiling at the forensic laboratory for DNA-research (ISO 17025) and all cell lines matched for 100% with the known profile [31]. All cell lines were cultivated in RPMI-1640 medium containing L-glutamine, 10% fetal bovine serum (FBS) (Invitrogen, Carlsbad, CA, USA) and streptomycin/penicillin (Sigma-Aldrich, St. Louis, MO, USA) at 5% CO_2_ and 37 °C. Cells were maintained till approximately 80% confluence under sterile conditions. For harvesting, cells were washed twice with 1× PBS and incubated for approximately 5 min in 1× trypsin/EDTA solution in 1x PBS, whose activity was inhibited by the addition of serum containing medium following visual cell detachment. Cells were subsequently harvested and counted using the CountessTM Automated Cell Counter (Invitrogen, Paisley, UK). Aliquots of 2 × 10^7^ or 4 × 10^6^ cells were washed with 1× PBS and centrifuged at 1500 rpm to obtain cell pellets. Cell pellets were stored at −20 °C until use for MGL pull-downs or TMT labeling, respectively.

### 3.2. Lectins and Antibodies

Chimeric MGL-Fc was prepared as described previously [32]. For western blot staining the following antibody were used: c-Met mouse mAb (3D4, Thermo Fisher Scientific, Waltham, MA, USA, concentration 1.5 ug/mL), phospho-Met rabbit mAb (Tyr1234/1235) (D26, Cell Signaling Scientific, Danvers, MA, USA, dilution 1:1000). For detection of c-Met, a secondary antibody goat anti-mouse immunoglobulins/HRP (Agilent Dako, Santa Clara, CA, USA, dilution 1:2000) was used, while for phospho-Met, swine anti-rabbit immunoglobulins/HRP (Agilent Dako, dilution 1:2000). For lectin blot, MGL-Fc was targeted with a secondary antibody peroxidase-conjugated goat anti-human IgG-Fcγ (Jackson Immuno Research, West Grove, PA, USA, dilution 1:1500).

### 3.3. Pull-Down Assay and PNGase F Treatment

Protein extracts were obtained as described before [33]: cell pellets were incubated for 20 min on ice in lysis buffer (10 mM triethanolamine pH 8.2, 150 mM NaCl, 1 mM MgCl_2_, 1 mM CaCl_2_ and 1% (volume/volume) Triton X-100, containing EDTA-free protease inhibitor (Roche Diagnostics, Almere, Netherlands)). Protein quantification was performed using the BCA assay (BCA Protein Assay Kit, Pierce™, Thermo Fisher Scientific, Waltham, MA, USA), following the manufacturer’s instructions. MGL ligands were pulled down from 1 mg of protein extracts with or without prior treatment with PNGase F PRIME (N-Zyme Scientifics, Doylestown, PA, USA, concentration 0.1 ug/mL) overnight at 37 °C. Two µg of chimeric MGL-Fc, coupled to 50 µL Dynabeads protein G (Invitrogen) were used as previously described [33]. Following washing, the elution of specific ligands was performed using 100 mM EDTA.

### 3.4. SDS-PAGE and Western/Lectin Blot

Protein extracts and MGL pull-down samples were separated by SDS-PAGE (4–15% Mini-PROTEAN^®^ TGX Stain-Free™ Protein Gels, Bio-Rad) and transferred to a PVDF membrane (Trans-Blot Turbo Mini PVDF Transfer Packs, Bio-Rad, Hercules, CA, USA). 5% bovine serum albumin (Sigma-Aldrich) in 0.1% phosphate-buffered saline with Tween-20 (Sigma-Aldrich) (PBS-T) was used to block the blots for 1 h. Immunoblotting was performed with specific antibodies in BSA 1% PBS-T, followed by peroxidase-conjugated secondary antibodies. For lectin blots, 5% BSA in TSM buffer (20 mM Tris-HCl, pH 7.4, 150 mM NaCl, 1 mM CaCl_2_ and 2 mM MgCl_2_) was used as blocking buffer, and incubation with the lectin was performed in BSA 1% TSM. The following washes and incubation with peroxidase-conjugated secondary antibodies were performed in TSM with 0.1% Tween-20. Immunodetection was done by enhanced chemiluminescence (ECL) using Clarity Western ECL substrate (Bio-Rad) and an Amersham Imager 600 (Cytiva, Marlborough, United States).

### 3.5. SDS-PAGE and NanoLC-MS/MS Analysis

For sample clean-up, a short SDS-PAGE run (NuPAGE™ 4–12% Bis-Tris Protein Gels, Thermo Fisher Scientific) of the samples obtained from the MGL pull-downs after PNGase F treatment was performed. Gels were stained with SimplyBlue™ Safe Stain (Invitrogen) for 1 h at room temperature (RT) and washed with distilled water for 3 h. Bands corresponding to the whole lane were cut from the gel, and the proteins were then subjected to reduction with dithiothreitol (10 mM), alkylation with iodoacetamide (50 mM) and in-gel trypsin digestion with trypsin (Worthington Enzymes Lakewood, NJ, United States), using a Proteineer DP digestion robot (Bruker, Billerica, MA, United States) [33].

Tryptic peptides were separated by online C18 nano- High Performance Liquid Chromatography (HPLC)-MS/MS with an Easy nLC 1000 gradient HPLC system (Thermo, Bremen, Germany) coupled to an Orbitrap Fusion LUMOS mass spectrometer (Thermo), as previously described [33]. Briefly, fractions were loaded onto a homemade precolumn and eluted via a homemade analytical nano-HPLC column (for 20 min), followed by electrospray injection into the mass spectrometer. The MS was operated in data-dependent MS/MS (top-10 mode) with a normalized collision energy of 32% and recording of the MS^2^ spectrum in the Orbitrap (parameters specified in [33]). For protein identification, raw data was converted to mzXML using Proteowizard software. Peptide and protein identification as well as the after statistical validation were performed in Trans Proteomics Pipeline version 5.1.0 using included software pipeline: X! Tandem Jackhammer TPP (version 2013.06.15.1-LabKey, Insilicos, ISB) search engine, PeptideProphet, and ProteinProphet. The parameters were set as follows: precursor mass error of 10 ppm, fragment mass error of 0.04 Da, carbamidomethyl (Cys) and oxidation (Met) as fixed and variable modifications, respectively. All results were filtered for false discovery rate (FDR) threshold of 1% as well as a minimum of two per protein. Data extraction and table generation was done using R version 3.4.4.

### 3.6. Quantitative Proteomics Using TMT Labeling

Cell lysis, digestion and TMT labeling was performed as described [34]. Cellular extract from 4 × 10^6^ HCT116, HT29, and LS174T cells were prepared in triplicate by a 4 min incubation at 95 °C in SDS lysis buffer (5% SDS, 100 mM Tris-HCl pH 7.6). Protein concentration was determined by BCA assay. 100 µg of protein was used for subsequent reduction with 5 mM TCEP, alkylation with 15 mM iodoacetamide and quenching with 10 mM DTT. Protein lysates were cleaned by methanol-chloroform precipitation. The resulting protein pellets were resuspended in 40 mM Hepes (pH 8.4) and incubated with 10 µg trypsin O/N at 37 °C. Peptide concentration was measured with BCA assay. 10 µg of each of the 9 peptide preparations was dissolved in 25 µL of 40 mM Hepes (pH 8.4) and incubated with 40 µg of one of the 9 amino reactive TMT10plex Label Reagents (126 to 130, Thermo Scientific, Lot #UG282327) for 1 h at RT. Excess TMT label was quenched by incubation with 6 μL 5% hydroxylamine for 15 min at RT. The 9 labeled peptide samples were then mixed, freeze-dried and measured using MultiNotch MS^3^ procedure [35].

TMT-labeled peptides were dissolved in 0.1% formic acid and subsequently analyzed by online C18 nano-HPLC MS/MS with a system consisting of an Easy nLC 1200 gradient HPLC system (Thermo, Bremen, Germany), and an Orbitrap Fusion LUMOS mass spectrometer (Thermo). Fractions were injected onto a homemade precolumn (100 μm × 15 mm; Reprosil-Pur C18-AQ 3 μm, Dr Maisch, Ammerbuch, Germany) and eluted via a homemade analytical nano-HPLC column (50 cm × 75 μm; Reprosil-Pur C18-AQ 1.9 μm). The analytical column temperature was maintained at 50 °C with a PRSO-V2 column oven (Sonation, Biberach, Germany). The gradient was run from 5% to 30% solvent B (20/80/0.1 water/acetonitrile/formic acid (FA) *v*/*v*) in 240 min. The nano-HPLC column was drawn to a tip of ~5 μm and acted as the electrospray needle of the MS source. The LUMOS mass spectrometer (Thermo) was set to use the MultiNotch MS^3^-based TMT method [35]. The MS spectrum was recorded in the Orbitrap (resolution 120,000; m/z range 400–1500; automatic gain control (AGC) target 2 × 10^5^; maximum injection time 50 ms). Dynamic exclusion was after *n* = 1 with an exclusion duration of 60 s with a mass tolerance of 10 ppm. Charge states 2–4 were included Precursors for MS^2^/MS^3^ analysis were selected using a TopSpeed of 3 sec. MS^2^ analysis consisted of collision-induced dissociation (quadrupole ion trap analysis; AGC 1 × 10^4^; normalized collision energy (NCE) 35; maximum injection time 50 ms). The isolation window for MS/MS was 0.7 Da. Following acquisition of each MS^2^ spectrum, the MultiNotch MS^3^ spectrum was recorded using an isolation window for MS^3^ of 2 Da. MS^3^ precursors were fragmented by high energy collision-induced dissociation (HCD) and analyzed using the Orbitrap, NCE 65; AGC 1 × 10^5^; maximum injection time 105 ms, resolution 60,000).

In a post-analysis process, raw data were first converted to peak lists using Proteome Discoverer version 2.4 (Thermo Electron, Waltham, MA, United States), and then submitted to the Uniprot Homo sapiens minimal database (20205 entries), using Mascot v. 2.2.04 (www.matrixscience.com) for protein identification. Mascot searches were done with 10 ppm and 0.02 Da deviation for precursor and fragment mass, respectively, and trypsin enzyme was specified. Methionine oxidation and acetyl (Protein N-term) were set as variable modifications and Carbamidomethyl (C) was set as a static modification. Peptides with an FDR < 1% were accepted. The TMT ratio from the MultiNotch MS^3^ spectra were used for quantification using Proteome Discoverer 2.4.

### 3.7. Data Availability

The mass spectrometry proteomics data have been deposited to the ProteomeXchange Consortium via the PRIDE [36] partner repository with the dataset identifier PXD020344.

## 4. Conclusions

Notwithstanding the importance of the Tn antigen for the binding to MGL in CRC [15,21,37], our current study demonstrates a hitherto unrecognized notable contribution of protein *N*-glycosylation for the binding of MGL to glycoproteins of CRC cell lines. This should be considered in future investigations aiming to understand the responses in immune cells, but also cancer cells, following interaction of MGL with its ligands. In fact, a variety of MGL mediated responses have been described. On the one hand, activation of MGL on DC’s by synthetic glycopeptides carrying Tn structures (e.g., from CD45, CD43 or MUC1), showed an immunosuppressive response in cancer [38]. On the other hand, the MGL binding to Tn-bearing CD45 on T cell leukemia cells induced cell death [13]. Moreover, MGL signal transmission and outcome is dependent on the type of glycan structure [39] as well as the peptide backbone binding to the secondary binding site in the MGL CRD [14]. For this reason, we believe that the identification of MGL ligands will help to understand whether MGL binding to cancer cells induce receptor-specific signaling thereby promoting or reducing cell survival.

With the identification of more than 6000 proteins through our proteomics study, we gained more insights into the MGL-binding phenotype of HCT116 and HT29 compared to LS174T. First, we found the major MGL-binding proteins from HT29 and HCT116 cells were found at comparable levels in LS174T cells. Moreover, this analysis ruled out the major role of mucins as MGL binders in CRC cell lines, in contrast with many MGL investigations on CRC tissues [37] and other cancer types [23]. Even though the higher levels of GALNT3 in HT29 could partly explain the high MGL binding to this cell line, the involvement of other glycosylation enzymes in the specific glycotope on the MGL ligands in HT29 and HCT116 warrants further investigation. Our study indicates that downstream targets of CDX-2 could be good candidates.

## Supplementary Materials

Supplementary materials can be found at https://www.mdpi.com/1422-0067/21/15/5522/s1. Supplemental Figure S1: MGL staining of MGL-binding proteins from HCT116, HT29, and LS174T following *N*-glycan release. Supplemental Figure S2: c-Met levels and activation in HCT116, HT29, and LS174T. Supplemental Figure S3: Volcano plots of binary comparisons of protein abundances in the three CRC cell lines (HCT116, HT29, and LS174T) based on quantitative proteomics analysis. Supplemental Figure S4: Proteins observed at different levels in the high-MGL-binding cell lines (HT29 and HCT116) compared to the low MGL-binding cell line. Table S1: Release of *N*-glycans reduces the MGL-binding of proteins from CRC cell lines. Table S2: Raw data of comparative quantitative proteomics with TMT labeling on HCT116, HT29, and LS174T. The binary comparison of the protein abundance is shown. Table S3: Binary abundance ratio of the major cell surface MGL-binding proteins in HCT116 and HT29 cells compared to LS174T.

## Abbreviations

MGL	Macrophage galactose-type C-type lectin
CRC	Colorectal cancer
TACA	Tumor-Associated Carbohydrate Antigens
CRD	Carbohydrate recognition domain
TMT	Tandem Mass Tag
Fc	Fragment crystallizable
mAb	Monoclonal antibody
HRP	Horseradish peroxidase
LC	Liquid chromatography
MS	Mass Spectrometry

## References

[1] Munkley J., Elliott D.J. (2016). Hallmarks of glycosylation in cancer. Oncotarget.

[2] Stowell S.R., Ju T., Cummings R.D. (2015). Protein glycosylation in cancer. Annu. Rev. Pathol..

[3] Varki A. (2017). Biological roles of glycans. Glycobiology.

[4] Blanas A., Sahasrabudhe N.M., Rodriguez E., van Kooyk Y., van Vliet S.J. (2018). Fucosylated Antigens in Cancer: An Alliance toward Tumor Progression, Metastasis, and Resistance to Chemotherapy. Front. Oncol..

[5] Holst S., Wuhrer M., Rombouts Y. (2015). Glycosylation characteristics of colorectal cancer. Adv. Cancer Res..

[6] Pinho S.S., Reis C.A. (2015). Glycosylation in cancer: Mechanisms and clinical implications. Nat. Rev. Cancer.

[7] Beckwith D.M., Cudic M. (2020). Tumor-associated O-glycans of MUC1: Carriers of the glyco-code and targets for cancer vaccine design. Semin. Immunol..

[8] Cervoni G.E., Cheng J.J., Stackhouse K.A., Heimburg-Molinaro J., Cummings R.D. (2020). O-glycan recognition and function in mice and human cancers. Biochem. J..

[9] Silva M.L.S. (2019). Lectin biosensors in cancer glycan biomarker detection. Adv. Clin. Chem..

[10] Ju T., Wang Y., Aryal R.P., Lehoux S.D., Ding X., Kudelka M.R., Cutler C., Zeng J., Wang J., Sun X. (2013). Tn and sialyl-Tn antigens, aberrant O-glycomics as human disease markers. Proteom. Clin. Appl..

[11] Hirano K., Matsuda A., Shirai T., Furukawa K. (2014). Expression of LacdiNAc groups on N-glycans among human tumors is complex. Biomed. Res. Int..

[12] Jin C., Kenny D.T., Skoog E.C., Padra M., Adamczyk B., Vitizeva V., Thorell A., Venkatakrishnan V., Linden S.K., Karlsson N.G. (2017). Structural Diversity of Human Gastric Mucin Glycans. Mol. Cell Proteom..

[13] van Vliet S.J., Gringhuis S.I., Geijtenbeek T.B., van Kooyk Y. (2006). Regulation of effector T cells by antigen-presenting cells via interaction of the C-type lectin MGL with CD45. Nat. Immunol..

[14] Marcelo F., Supekar N., Corzana F., van der Horst J.C., Vuist I.M., Live D., Boons G.P.H., Smith D.F., van Vliet S.J. (2019). Identification of a secondary binding site in human macrophage galactose-type lectin by microarray studies: Implications for the molecular recognition of its ligands. J. Biol. Chem..

[15] Lenos K., Goos J.A., Vuist I.M., den Uil S.H., Delis-van Diemen P.M., Belt E.J., Stockmann H.B., Bril H., de Wit M., Carvalho B. (2015). MGL ligand expression is correlated to BRAF mutation and associated with poor survival of stage III colon cancer patients. Oncotarget.

[16] Pirro M., Rombouts Y., Stella A., Neyrolles O., Burlet-Schiltz O., van Vliet S.J., de Ru A.H., Mohammed Y., Wuhrer M., van Veelen P.A. (2020). Characterization of Macrophage Galactose-type Lectin (MGL) ligands in colorectal cancer cell lines. Biochim. Biophys. Acta Gen. Subj..

[17] Holst S., Deuss A.J., van Pelt G.W., van Vliet S.J., Garcia-Vallejo J.J., Koeleman C.A., Deelder A.M., Mesker W.E., Tollenaar R.A., Rombouts Y. (2016). N-glycosylation Profiling of Colorectal Cancer Cell Lines Reveals Association of Fucosylation with Differentiation and Caudal Type Homebox 1 (CDX1)/Villin mRNA Expression. Mol. Cell Proteom..

[18] Madunic K., Zhang T., Mayboroda O.A., Holst S., Stavenhagen K., Jin C., Karlsson N.G., Lageveen-Kammeijer G.S.M., Wuhrer M. (2020). Colorectal cancer cell lines show striking diversity of their O-glycome reflecting the cellular differentiation phenotype. Cell Mol. Life Sci..

[19] Parizadeh S.M., Jafarzadeh-Esfehani R., Fazilat-Panah D., Hassanian S.M., Shahidsales S., Khazaei M., Parizadeh S.M.R., Ghayour-Mobarhan M., Ferns G.A., Avan A. (2019). The potential therapeutic and prognostic impacts of the c-MET/HGF signaling pathway in colorectal cancer. Iubmb Life.

[20] Berg K.C.G., Eide P.W., Eilertsen I.A., Johannessen B., Bruun J., Danielsen S.A., Bjornslett M., Meza-Zepeda L.A., Eknaes M., Lind G.E. (2017). Multi-omics of 34 colorectal cancer cell lines—A resource for biomedical studies. Mol. Cancer.

[21] Sahasrabudhe N.M., Lenos K., van der Horst J.C., Rodriguez E., van Vliet S.J. (2018). Oncogenic BRAFV600E drives expression of MGL ligands in the colorectal cancer cell line HT29 through N-acetylgalactosamine-transferase 3. Biol. Chem..

[22] Moriwaki K., Noda K., Furukawa Y., Ohshima K., Uchiyama A., Nakagawa T., Taniguchi N., Daigo Y., Nakamura Y., Hayashi N. (2009). Deficiency of GMDS leads to escape from NK cell-mediated tumor surveillance through modulation of TRAIL signaling. Gastroenterology.

[23] Beatson R., Maurstad G., Picco G., Arulappu A., Coleman J., Wandell H.H., Clausen H., Mandel U., Taylor-Papadimitriou J., Sletmoen M. (2015). The Breast Cancer-Associated Glycoforms of MUC1, MUC1-Tn and sialyl-Tn, Are Expressed in COSMC Wild-Type Cells and Bind the C-Type Lectin MGL. PLoS ONE.

[24] Kurze A.K., Buhs S., Eggert D., Oliveira-Ferrer L., Muller V., Niendorf A., Wagener C., Nollau P. (2019). Immature O-glycans recognized by the macrophage glycoreceptor CLEC10A (MGL) are induced by 4-hydroxy-tamoxifen, oxidative stress and DNA-damage in breast cancer cells. Cell Commun. Signal..

[25] Saeland E., Belo A.I., Mongera S., van Die I., Meijer G.A., van Kooyk Y. (2012). Differential glycosylation of MUC1 and CEACAM5 between normal mucosa and tumour tissue of colon cancer patients. Int. J. Cancer.

[26] Boyd M., Hansen M., Jensen T.G., Perearnau A., Olsen A.K., Bram L.L., Bak M., Tommerup N., Olsen J., Troelsen J.T. (2010). Genome-wide analysis of CDX2 binding in intestinal epithelial cells (Caco-2). J. Biol. Chem..

[27] Lauc G., Essafi A., Huffman J.E., Hayward C., Knezevic A., Kattla J.J., Polasek O., Gornik O., Vitart V., Abrahams J.L. (2010). Genomics meets glycomics-the first GWAS study of human N-Glycome identifies HNF1alpha as a master regulator of plasma protein fucosylation. PLoS Genet..

[28] Pinto R., Barros R., Pereira-Castro I., Mesquita P., da Costa L.T., Bennett E.P., Almeida R., David L. (2015). CDX2 homeoprotein is involved in the regulation of ST6GalNAc-I gene in intestinal metaplasia. Lab. Invest..

[29] Holst S., Wilding J.L., Koprowska K., Rombouts Y., Wuhrer M. (2019). N-Glycomic and Transcriptomic Changes Associated with CDX1 mRNA Expression in Colorectal Cancer Cell Lines. Cells.

[30] Conze T., Carvalho A.S., Landegren U., Almeida R., Reis C.A., David L., Soderberg O. (2010). MUC2 mucin is a major carrier of the cancer-associated sialyl-Tn antigen in intestinal metaplasia and gastric carcinomas. Glycobiology.

[31] Westen A.A., Kraaijenbrink T., Robles de Medina E.A., Harteveld J., Willemse P., Zuniga S.B., van der Gaag K.J., Weiler N.E., Warnaar J., Kayser M. (2014). Comparing six commercial autosomal STR kits in a large Dutch population sample. Forensic. Sci. Int. Genet..

[32] van Vliet S.J., van Liempt E., Saeland E., Aarnoudse C.A., Appelmelk B., Irimura T., Geijtenbeek T.B., Blixt O., Alvarez R., van Die I. (2005). Carbohydrate profiling reveals a distinctive role for the C-type lectin MGL in the recognition of helminth parasites and tumor antigens by dendritic cells. Int. Immunol..

[33] Pirro M., Schoof E., van Vliet S.J., Rombouts Y., Stella A., de Ru A., Mohammed Y., Wuhrer M., van Veelen P.A., Hensbergen P.J. (2019). Glycoproteomic Analysis of MGL-Binding Proteins on Acute T-Cell Leukemia Cells. J. Proteome Res..

[34] Paulo J.A., Gygi S.P. (2017). Nicotine-induced protein expression profiling reveals mutually altered proteins across four human cell lines. Proteomics.

[35] McAlister G.C., Nusinow D.P., Jedrychowski M.P., Wuhr M., Huttlin E.L., Erickson B.K., Rad R., Haas W., Gygi S.P. (2014). MultiNotch MS3 enables accurate, sensitive, and multiplexed detection of differential expression across cancer cell line proteomes. Anal. Chem..

[36] Vizcaino J.A., Csordas A., Del-Toro N., Dianes J.A., Griss J., Lavidas I., Mayer G., Perez-Riverol Y., Reisinger F., Ternent T. (2016). 2016 update of the PRIDE database and its related tools. Nucleic Acids Res..

[37] Saeland E., van Vliet S.J., Backstrom M., van den Berg V.C., Geijtenbeek T.B., Meijer G.A., van Kooyk Y. (2007). The C-type lectin MGL expressed by dendritic cells detects glycan changes on MUC1 in colon carcinoma. Cancer Immunol. Immunother..

[38] van Vliet S.J., Bay S., Vuist I.M., Kalay H., Garcia-Vallejo J.J., Leclerc C., van Kooyk Y. (2013). MGL signaling augments TLR2-mediated responses for enhanced IL-10 and TNF-alpha secretion. J. Leukoc Biol..

[39] Diniz A., Coelho H., Dias J.S., van Vliet S.J., Jimenez-Barbero J., Corzana F., Cabrita E.J., Marcelo F. (2019). The Plasticity of the Carbohydrate Recognition Domain Dictates the Exquisite Mechanism of Binding of Human Macrophage Galactose-Type Lectin. Chemistry.

