# Supplementary material for: N-Glycoproteins Have a Major Role in MGL Binding to Colorectal Cancer Cell Lines: Associations with Overall Proteome Diversity
Source: Int J Mol Sci. 2020 Aug 1;21(15):5522. doi: 10.3390/ijms21155522 (PMC7432225; doi:10.3390/ijms21155522)
Supplement: Supplementary file 1 [file ijms-21-05522-s001.zip › ijms-868080-supplementary/Pirro et al_IJMS_supplemental figures.docx.docx]

***Supplemental data***

***N*-glycoproteins have a major role in MGL binding to colorectal cancer cell lines: associations with overall proteome diversity**

**Martina Pirro^1^, Yassene Mohammed^1^, Sandra J. van Vliet^2^, Yoann Rombouts^3^, Agnese Sciacca^1^, Arnoud H. de Ru^1^, George M. C. Janssen^1^, Rayman T. N. Tjokrodirijo^1^, Manfred Wuhrer^1^, Peter A. van Veelen^1^, Paul J. Hensbergen^1^***

^1^ Center for Proteomics and Metabolomics, Leiden University Medical Center, Leiden, The Netherlands

^2^ Amsterdam UMC, Vrije Universiteit Amsterdam, dept. of Molecular Cell Biology and Immunology, Cancer Center Amsterdam, Amsterdam Infection & Immunity Institute, Amsterdam, the Netherlands

^3^ Institut de Pharmacologie et de Biologie Structurale, Université de Toulouse, CNRS, UPS, Toulouse, France

***** To whom correspondence should be addressed:

P.J. Hensbergen

Center for Proteomics and Metabolomics

Leiden University Medical Center

PO Box 9600, 2300 RC Leiden, The Netherlands;

Email: P.J.Hensbergen@lumc.nl;

Tel.: +31-71-5266394, Fax: +31-71-5266907

***Supplemental figure 1: MGL staining of MGL-binding proteins from HCT116, HT29 and LS174T following N-glycan release.*** *MGL lectin blot of MGL pull-downs, performed without (-) or with PNGase F treatment (A, B). The N-glycans were released either* ***A****fter (A) or* ***B****efore (B) performing MGL pull-downs. The lectin blots represents two out of three biological replicates (The first replicate is shown in figure 1). Red arrows indicate major stained bands. M.W.: Molecular weight.*


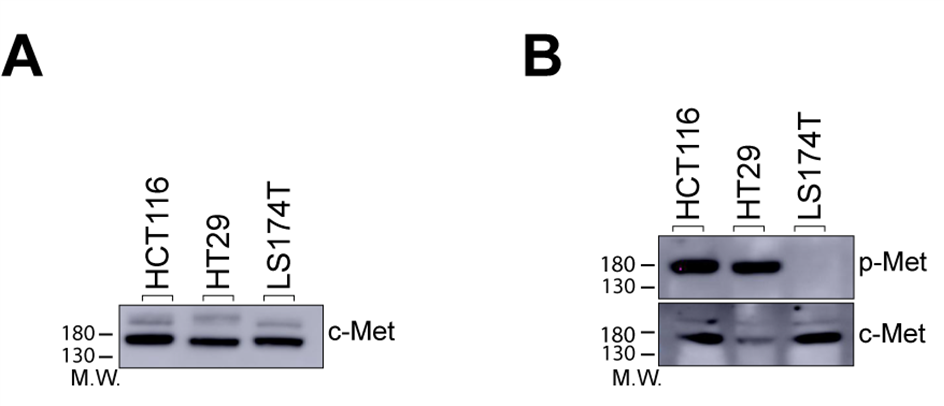


***Supplemental figure 2: c-Met levels and activation in HCT116, HT29 and LS174T. A)*** *Western blot analysis of c-Met protein levels in the three CRC cell lines.* ***B)*** *Western blot analysis of c-Met activation (Tyrosine 1234/1235 phosphorylation, p-Met) in the three CRC cell lines under baseline culture conditions. M.W.: Molecular weight.*


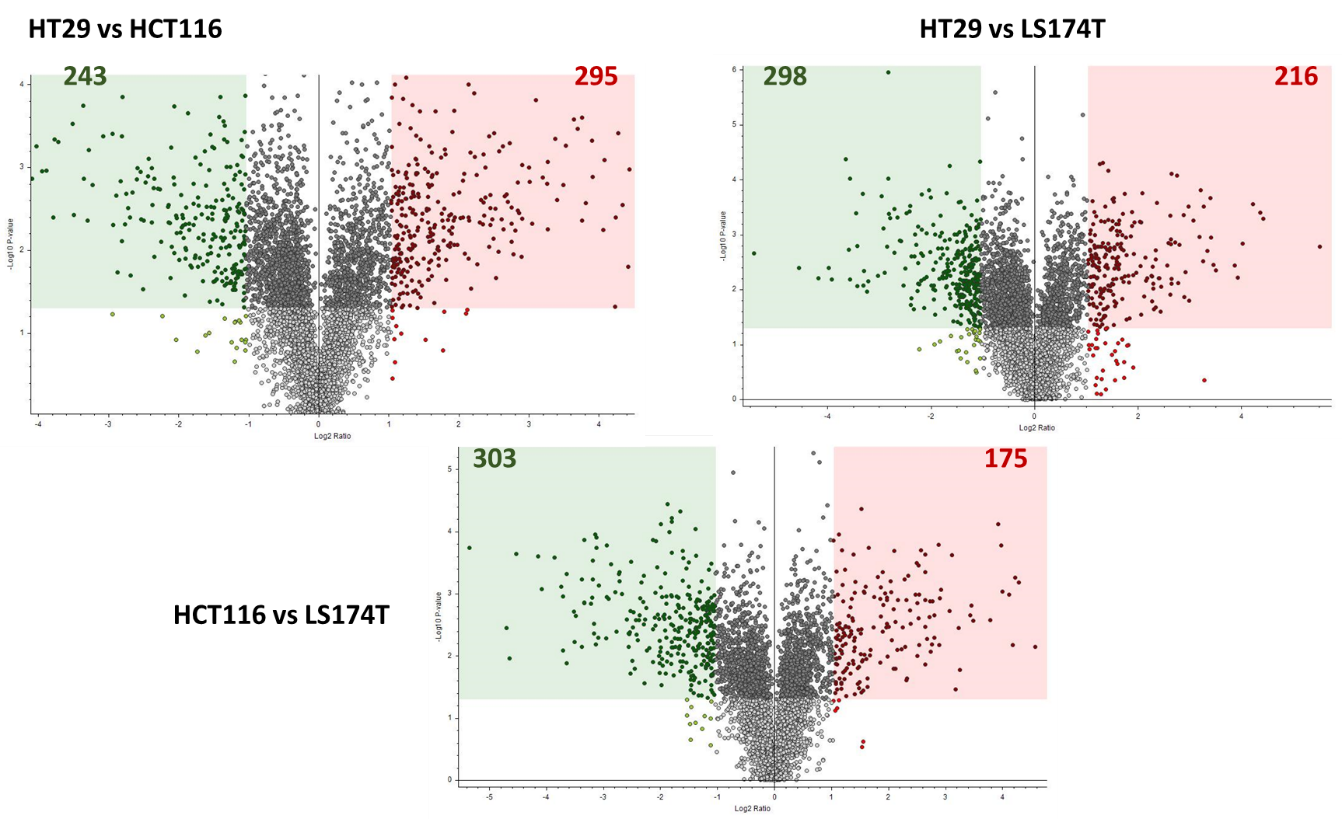


***Supplemental figure 3: Volcano plots of binary comparisons of protein abundances in the three CRC cell lines (HCT116, HT29 and LS174T) based on quantitative proteomics analysis.*** *Proteins at higher abundance (log_2_ ratio>1, p<0.05) are shown in red, the ones at lower abundance (log_2_ ratio<-1, p<0.05) in green.*

***
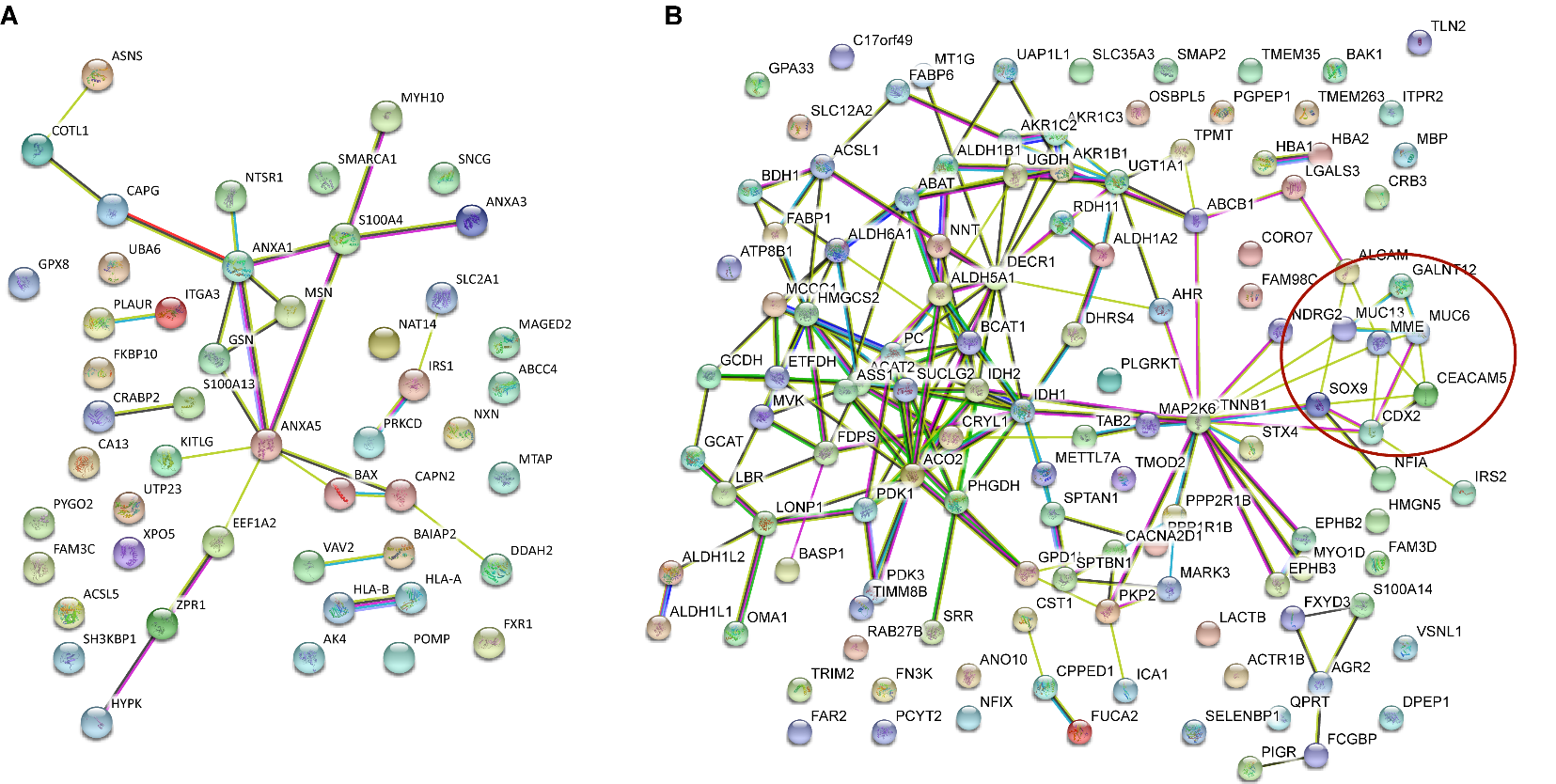
******Supplemental figure 4: Proteins observed at different levels in the high MGL binding cell lines (HT29 and HCT116) compared to the low MGL-binding cell line.*** *Functional protein association networks of proteins higher (A) and lower (B) in the high MGL binding cell lines analysed by STRING (version 11.0). The cluster of mucins and CDX-2 is highlighted by the red ellipse.The empty nodes represent proteins of unknown 3D structure, while filled nodes have a known or predicted 3D structure. Edges represent protein-protein interactions either as known (light blue = from database, purple = experimental) or predicted (green = gene neighborhood, red = gene fusion, blue = gene co-occurrence). Other associations are either derived from text mining (yellow), co-expression (black) or protein homology (grey).*
